# Computer Alloy Design of Ti Modified Al-Si-Mg-Sr Casting Alloys for Achieving Simultaneous Enhancement in Strength and Ductility

**DOI:** 10.3390/ma16010306

**Published:** 2022-12-28

**Authors:** Shaoji Zhang, Wang Yi, Jing Zhong, Jianbao Gao, Zhao Lu, Lijun Zhang

**Affiliations:** 1 State Key Laboratory of Powder Metallurgy, Central South University, Changsha 410083, China; 2 Guangxi Key Laboratory of Information Materials, School of Material Science and Engineering, Guilin University of Electronic Technology, Guilin 530004, China

**Keywords:** casting aluminum alloy, alloy design, CALPHAD, machine learning, Ti modification

## Abstract

In this paper, an efficient design of a Ti-modified Al-Si-Mg-Sr casting alloy with simultaneously enhanced strength and ductility was achieved by integrating computational thermodynamics, machine learning, and key experiments within the Bayesian optimization framework. Firstly, a self-consistent Al-Si-Mg-Sr-Ti quinary thermodynamic database was established by the calculation of phase diagram method and verified by key experiments. Based on the established thermodynamic database, a high-throughput Scheil-Gulliver solidification simulation of the A356-0.005Sr alloy with different Ti contents was carried out to establish the “composition-microstructure” quantitative relationship of the alloy. Then, by combining the computational thermodynamic, machine learning, and experimental data within the Bayesian optimization framework, the relationship “composition/processing-microstructure-properties” of A356-0.005Sr with different Ti contents was constructed and validated by the key experiments. Furthermore, the optimum alloy composition of the Ti-modified A356-0.005Sr casting alloy was designed based on this integration method with the Bayesian optimization framework and verified by the experiments. It is anticipated that the present integration method may serve as a general one for the efficient design of casting alloys, especially in the high-dimensional composition space.

## 1. Introduction

Al-Si-Mg casting alloys are widely used in the automobile and building industries due to their excellent castability, corrosion resistance, good mechanical properties, and low density [1]. However, the mechanical properties of Al-Si-Mg casting alloys are still limited by the negative factors in the as-cast microstructure, including the coarse primary (Al) and plate-like eutectic (Si). In general, the mechanical properties and performance of Al-Si-Mg casting alloys can be enhanced by modifying the microstructure (such as grain refinement, precipitation hardening, solid solution hardening, and so on) through, e.g., the addition of alloying/micro-alloying elements [2]. For instance, Ti and Sr are two common alloying elements in Al-Si-Mg casting alloys. It is generally believed that while Sr addition can modify the morphology of eutectic (Si) from plate-like into finer fibrous [3,4,5]; Ti addition can produce Al_3_Ti as heterogeneous nucleation sites for primary (Al) to realize the refinement of (Al) grains [6,7]. Additionally, Schumacher et al. [8,9,10] found that Ti could form an Al_3_Ti thin layer on the surface of TiB_2_ and improve the nucleation efficiency of the TiB_2_; StJohn et al. [11,12,13,14] found that in aluminum alloys containing TiB_2_, the grain size could be effectively reduced with the addition of Ti content, and the grain size was linearly related to 1/Q (Q is the growth restriction factor) in a certain composition range, but the element Ti had little effect on the SDAS (secondary dendrite arm spacing) of aluminum alloys. To achieve the best comprehensive mechanical properties of the Ti/Sr-additional Al-Si-Mg casting alloys, it is necessary to obtain the optimal additional amounts of Ti and Sr. Lipiński [15,16,17] performed a series of experimental studies on the effect of Sr, Ti, and B in the form of powder and/or preliminary alloys on the microstructure and mechanical properties of the Al-9Si-Mg alloy. His results indicated that there was higher strength in the heat-treated Al-9Si-Mg alloy with preliminary alloy, while the modified elements showed a significant positive effect on the mechanical properties of the alloy. Meanwhile, the order of element addition also affected the alloy properties. Samuel et al. [18] experimentally investigated the effect of Ti addition on the impact toughness of Sr-modified A356.2 alloys by using an Al–10%Ti master alloy. They found that the alloy reached its highest value of total absorbed energy after a T6 heat treatment when 0.04% Ti was added. However, it is challenging to design the optimal amount of additional elements for Al-Si-Mg alloys by using only the trial-and-error experimental method, which is also time-consuming and costly. Moreover, it is worth noting that Chen and Fortier [19] point out that when Ti exceeds 0.1 wt.% in the A356 alloy, problems such as feeding blockage and wormhole defects appear due to the generation of the TiAlSi phase. According to their computational thermodynamics (CT) results, Li [20] concluded that good grain refinement and casting quality can be achieved when the Ti content is lower than ca. 0.15 wt.% in the Al-7Si alloy. Therefore, the optimal amount of Ti addition in Al-Si-Mg alloys in the low addition range is more worthy of study.

Under this situation, theoretical approaches can serve as important alternatives. For the past decades, some theoretical alloy design approaches, such as CT [21,22,23], phase-field simulation [24,25], computational kinetics [26,27], first-principles calculation [28,29], and machine learning (ML) [30,31], have been successfully used to design a variety of alloys. The CALPHAD (CALculation of PHAse Diagram) technique in the framework of CT can be used to construct the non-equilibrium solidification diagram [21] for casting alloys based on the reliable thermodynamic database of the target alloys. With such a solidification diagram, the relationship between the composition and microstructure (here, only phase type and phase fraction are considered) can then be established. Moreover, ML, as an efficient alloy design method, can effectively construct complex relationships between the desired properties and predict the properties of unexplored alloys based on the established relationship [31,32]. A recent review by Yi et al. [2] proposed that combining ML and CT can speed up the accurate design of materials, e.g., casting alloys. Soon later, the same group of authors successfully applied the combinatorial method to design the optimal amount (i.e., 0.005 wt.%) of Sr addition in the A356 alloy [5] in an efficient manner. In order to verify the generality of such a combinatorial method, its applications in more real examples, e.g., Ti/Sr-modified Al-Si-Mg casting alloys, are needed.

For accurate CALPHAD predictions, a high-quality thermodynamic database is essential. Taking the Ti/Sr-modified Al-Si-Mg casting alloys, for instance, self-consistent thermodynamic descriptions for the quinary Al-Si-Mg-Sr-Ti system should be established a priori. However, there is always no ready-made reliable thermodynamic database for the multicomponent system in the literature, such as the quinary Al-Si-Mg-Sr-Ti system here.

ML is an effective technique for fitting the desired relationships and has been used with great success in materials design [33]. Very recently, ML was employed to construct the relationship between the microstructure and mechanical properties based on a small dataset in our research group [5]. However, the hyperparameters (i.e., the number of hidden layers), showing to have a large impact on the results, were only obtained by the trial-and-error method. Meanwhile, the next sampling scheme based on the model relied on human experience [5]. In order to get rid of the disadvantages, a Bayesian optimization framework, which combines the two steps of uncertainty assessment and decision-making, can be used to determine the next sampling scheme [34,35,36]. In this framework, the trained ML model is used to predict the values of random points considering the noise, and the uncertainty of the predicted results can be determined. The average expectation and uncertainty of the predicted results are then combined with the acquisition function to obtain the optimal composition of the target alloy. It is expected to avoid the influence of the hyperparameter selection on the results and implement the automatic recommendation of the next sampling, from which an efficient alloy design can be finally achieved.

Consequently, the major objectives of the present work are: (i) to establish the self-consistent thermodynamic database of the Al-Si-Mg-Sr-Ti system in the Al-rich region and validate its reliability by using the experimental data from the literature and/or the present work; (ii) to design the optimal Ti amount in the A356-0.005Sr alloy in the low addition range (i.e., ≤0.2 wt.% Ti) based on a Bayesian optimization framework integrating CT, ML, and key experiments and conduct the experimental validation.

## 2. Computational Methods

### 2.1. Thermodynamic Modeling

Since only the Al-rich Al-Si-Mg alloys are concerned in this work, the thermodynamic database of the target Al-Si-Mg-Sr-Ti system can be established by directly extrapolating from the thermodynamic descriptions of the Al-Si-Mg-Sr and Al-Si-Mg-Ti quaternary systems, together with key experimental validation. The thermodynamic descriptions for the Al-Si-Mg-Sr system recently established in our research group [5] were directly adopted here. While, for the Al-Si-Mg-Ti system, no thermodynamic descriptions have been reported in the literature. Thus, one needs to obtain the thermodynamic descriptions of the Al-Si-Mg-Ti system. There are four boundary ternary systems, i.e., Al-Si-Mg, Al-Si-Ti, Al-Mg-Ti, and Si-Mg-Ti, in the Al-Si-Mg-Ti quaternary system. Considering the fact that (i) we only focus on the design of Al-rich Al-Si-Mg alloys and (ii) the thermodynamic descriptions of the Al-Mg-Ti and Si-Mg-Ti ternary systems are lacking in the literature, the thermodynamic descriptions for the Al-Si-Mg-Ti system were also directly extrapolated from the boundary ternary Al-Si-Mg and Al-Si-Ti systems. To ensure compatibility with the adopted thermodynamic descriptions of the Al-Si-Mg-Sr system, the thermodynamic parameters of the Al-Si-Mg system assessed by Tang [37] were directly utilized in the Al-Si-Mg-Ti quaternary system. For the Al-Si-Ti system, the thermodynamic parameters reported by Li [20] were employed in the Al-Si-Mg-Ti quaternary system, except for those in the binary Al-Si system. That is because the thermodynamic descriptions of the Al-Si due to Li et al. [20] are inconsistent with those in the Al-Si-Mg system due to Tang et al. [37]. Here, the thermodynamic parameters of the Al-Si system reported by Tang et al. [37] were adopted to replace those in the Al-Si-Ti system. The updated thermodynamic descriptions of the ternary Al-Si-Ti system were verified and presented in Section S1 of the supplementary material. Figures S1–S3 show the comparison of the vertical section, isothermal section, and liquid phase surface projection below 900 °C calculated from the updated thermodynamic descriptions of the Al-Si-Ti ternary system with the experimental data from the literature, respectively. In order to visualize the process of database establishment, the schematic diagram is shown in Figure S4 in Section S2 of the supplementary material.

Following the general treatments in the development of CALPHAD thermodynamic databases [38,39], the thermodynamic models for the different phases involved in the target system should be defined a priori. The Gibbs energies for pure elements, including Al, Si, Mg, Sr, and Ti, were taken from the SGTE compilation by Dinsdale [40]. The solution phases, i.e., liquid, fcc, bcc, hcp, and diamond, can be described as substitution solution models [41,42]. The intermetallic compounds with negligible ternary solubility, i.e., Al_5_Ti_2_, Al_2_Ti, and Ti_3_Si, can be treated as stoichiometric ones. While for the intermetallic compounds with experimentally observed ternary solubility, the sub-lattice models can be applied [43]. Taking Al_3_Ti as an example, it can be modeled as (Ti, Al, Si)_0.75_(Ti, Al)_0.25_ [20] because of the limited solubility of Si. The analytical expressions for the Gibbs energies of the different phases in the present Al-Si-Mg-Sr-Ti system are presented in Section S2 of the supplementary material.

### 2.2. Scheil-Gulliver Solidification Simulations

The Scheil-Gulliver solidification model [44], which can effectively predict the solidification sequences and solidified microstructures of the alloy, is widely used in different alloys [5,21,45,46]. For example, Lu and Zhang [21] and Yi et al. [5] used the Scheil-Gulliver model to predict the solidification sequence, phase type, and phase fraction of aluminum alloys. Cheng et al. [46] utilized the Scheil-Gulliver model to predict the solidification sequences and the solidified microstructures in magnesium alloys. In the Scheil-Gulliver solidification mode [44], two general assumptions are applied, i.e., (i) that a local equilibrium exists over the solid-liquid interface at the solidification front and (ii) that the diffusion in the liquid phase is fast enough to reach equilibrium while no diffusion is considered in the solid phase.

Based on the established thermodynamic database of the quinary Al-Si-Mg-Sr-Ti system, the influence of element Ti on the solidification sequences and the solidification microstructures of A356-0.005Sr-*x*Ti (in wt.%) alloys was studied by the Scheil-Gulliver solidification mode. The process was completed on the high-throughput computing platform (Malac-Distmas) recently developed in our research group [47] coupled with the commercial software Pandat [48]. The solidification diagram of A356-0.005Sr with different Ti contents was constructed by integrating the simulation results, from which the quantitative relationship between the composition and solidification microstructure of the alloys was established.

### 2.3. Machine Learning Technique

**Figure 1:**
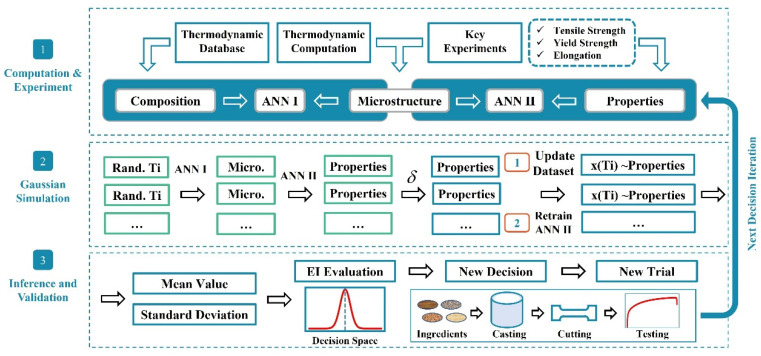
Machine learning training strategy diagram. Based on the thermodynamic database and experimental data, ANN I and ANN II artificial neural networks were used to establish the “composition-microstructure” and “microstructure-properties” relationships of the target alloys. Based on the Bayesian optimization process, a Gaussian simulation process was performed, and then the inference and validated results were obtained based on the simulation results. The new trail should be used in the next iteration.

The relation “composition/processing-microstructure-properties” is indispensable for materials design, as the inference and decision-making can be performed over the concerned composition space and processing windows towards the desired material properties. An artificial neural network can approximately approach complex function relations, which are generally non-linear and reside in the experimental observations, and make inference feasible once the neural architecture is properly selected and trained. Moreover, Bayesian optimization enables the screening of the most promising materials with the desired properties [36,49]. A Bayesian optimization framework is currently postulated to integrate the artificial neural network. A demonstration of the employed computational framework in this work is illustrated in Figure 1.

To begin with, the solidified microstructures for a series of alloys were predicted through Scheil-Gulliver solidification simulations based on the established thermodynamic database datasets, and the composition and corresponding solidified microstructures were then used as the dataset DS1. Subsequently, some key experiments of A356-0.005Sr-*x*Ti alloys were performed, and the experimental microstructures and mechanical properties were measured and used as the dataset DS2. An artificial neural network, namely ANN I, was employed for reconstructing the relation between the composition and solidified microstructure based on the DS1 dataset. Another artificial neural network, namely ANN II, was designed and trained over the dataset obtained from experimental characterization (hereinafter referred to as DS2), where the quantitative relations among the microstructure and mechanical properties, including ultimate tensile strength, yield strength, and elongation, were established.

Furthermore, Bayesian optimization was adopted for unearthing the most appropriate proposal for a composition of the optimal mechanical properties. A mimicry process was therefore introduced based on the ANN II model. That is, the routine for making inferences over random composition settings was carried out with the inferred properties as the products. Assuming that the confidence level of the experimental observation is δ, the inferred properties weighted with a random number in [1 − δ, 1 + δ] together with the composition setting and corresponding microstructure information were merged to form an augmented dataset. The trained ANN II was further re-trained and updated with the augmented dataset, and the inference towards the properties over the full composition range therefore proceeded. The purpose of this step is to obtain the augmented dataset, and each augmented dataset can be updated for a new ANN II, and the mimic data can be counted several times to obtain its mean and variance. The above-mentioned routine would be repeated, i.e., 1000 times, and a set of mimic observations would therefore be obtained. The statistics over the mimic observed dataset would be subsequently evaluated, i.e., expectations (mean value) and the corresponding variances (standard deviation).

The expected improvement (EI) [36,50], as the common acquisition function, could be calculated via

(1) $$EI\left(x\right)=\left(\mu \left(x\right)-f\left(x^{+}\right)-\xi \right)\cdot \Phi \left(Z\right)+\sigma \left(x\right)\cdot \phi \left(Z\right)$$

with

(2) $$x^{+}=\mathrm{argmax}f\left(x_{i}\right)$$

(3) $$\;Z=\frac{\mu \left(x\right)-f\left(x^{+}\right)-\xi }{\sigma \left(x\right)},$$

where $f(x_{i})$ is a function of the composition and the mean value obtained from all simulation processes. It refers to the surrogate model for the relation between the input *x* and output *y*. In this work, ANN II, which can predict the mechanical properties of the Ti-modified A356-0.005Sr alloys, was used as the surrogate model. $f(x^{+})$ is the best mechanical property so far under the condition $x^{+}$. μ(x) and σ(x) are the mean and standard deviation of the posterior distribution on *x* from the simulation processes, respectively. Φ(x) and ϕ(x) are the cumulative distribution function (CDF) and probability density function (PDF), respectively. The parameter *ξ* can be tuned to balance the trade-off between exploitation (i.e., the first term in Equation (1)) and exploration (i.e., the second term in Equation (1)).

Finally, the proposal for the most promising composition setting was obtained by maximizing the EI. The proposed composition should then be validated through practical experiment measurements. The whole process would be repeated multiple times when the concerned composition range is large, and the newly acquired data should be merged into the training dataset.

Artificial neural networks were built based on the PyTorch library [51], and OPTUNA [52] was employed for the artificial neural network hyperparameter optimization (including the number of hidden layers and nodes and the activation function). The Dataset DS1 for the ANN I model was obtained based on thermodynamic calculations using the present thermodynamic database. The fundamental dataset DS2 for ANN II was acquired through the key experiments in the present work. Moreover, the confidence level of the experimental data was assumed as ±5, i.e., δ=0.05.

## 3. Experimental Procedure

High-purity elemental raw materials (purity: 99.99 wt.%) of Al, Si, and Mg were employed. The elements Sr and Ti were added by master alloys Al-7.5%Sr and Al-5%Ti, respectively. It should be noted that all the compositions in this paper are expressed as weight percent unless otherwise stated. Each alloy corresponding to the nominal composition listed in Table 1 was melted in an electromagnetic inductive furnace under an argon gas atmosphere. In Table 1, all the experimental samples were divided into three series: (i) D series (3 samples) for validating the thermodynamic database, (ii) M series (6 samples) for constructing the experimental dataset of machine learning, and (iii) O series (only 1 sample) for validating the designed optimal composition. The extra 0.05 wt.% Mg was added considering that Mg is prone to evaporate during melting [21]. After homogenizing at 720 °C for 4 minutes and 750 °C for 4 minutes, respectively, the melt was poured into a cylindrical graphite mold (20 mm in diameter and 100 mm in height) preheated to 100 °C.

The chemical composition of each sample was measured by both inductively coupled plasma (ICP) and chemical analysis (CA) techniques. The plate-type tensile specimens were prepared by wire-electrode cutting, and all specimens were mechanically ground and further polished with oxide polishing suspension (OP-S) on an automatic mechanical polishing machine with a rotation speed of 250 rpm. The microstructures of all the samples were analyzed by optical microscopy (OM; Lecia-DM4500P, Leica Microsystems CMS GmbH, Wetzlar, Germany), back-scattered electron (BSE) imaging mode of scanning electron microscopy (SEM; JXA-8530, JEOL, Tokyo, Japan), and electron probe microanalysis (EPMA; JXA-8530, JEOL, Tokyo, Japan) techniques. The area fraction of relevant phases was measured using the Image-Pro Plus 6.0 metallographic analyzer. To reduce manual error, each specimen was measured in at least four different image views. The differential scanning calorimetry (DSC; STA 8000, PerkinElmer, Waltham, MA, USA) technique was utilized to determine the thermal effects of the alloys during heating and cooling in the temperature range of 400–700 °C at a speed of 10 K/min. A universal tensile machine (America Instron-3369) was used to test the tensile properties with a loading speed of 1 mm/min. Four identical tensile specimens of each alloy were tested at room temperature, and the mean values were accepted.

## 4. Results & Discussion

### 4.1. Thermodynamic Database of Quinary Al-Si-Mg-Sr-Ti System and Its Validation

#### 4.1.1. Quaternary Al-Si-Mg-Ti System

Since there is no literature report on the quaternary compound in the Al-Si-Mg-Ti quaternary system, the thermodynamic descriptions of the Al-Si-Mg-Ti quaternary system were first obtained by directly extrapolating the boundary ternary systems Al-Si-Mg and Al-Si-Ti [20,37]. The reliability of the thermodynamic descriptions was then verified by comparing the model-predicted results based on the thermodynamic descriptions with the experimental microstructure information available in the literature.

**Figure 2:**
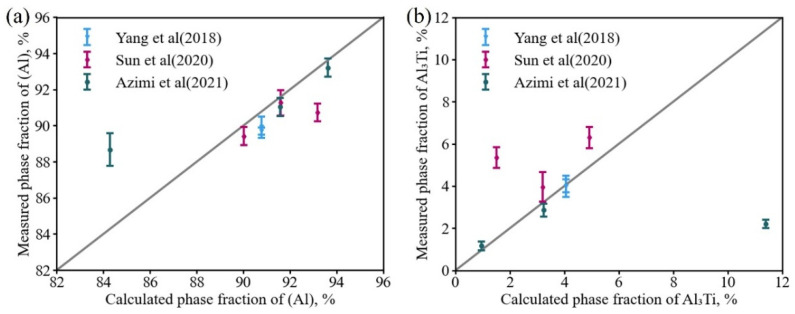
Comparison between the model-predicted fractions of (**a**) α-(Al), and (**b**) Al_3_Ti phase in as-casted Al-Si-Mg-Ti alloys and the experimental data from the literature [7,53,54]. In the plots, the calculated phase fraction is exactly the same as the measured phase fraction along the diagonal line.

Based on the microstructure images of the Al-Si-Mg-Ti alloy reported in the literature [7,53,54], the solidified phase fractions were counted by Image-Pro software and compared with the model-predicted results. The comparison results are shown in Figure 2. Figure 2a shows the comparison of the α-(Al) phase, while Figure 2b is the comparison of the Al_3_Ti phase. It should be noted that the model-predicted phase fraction matches the measured results exactly along the diagonal dashed line. As can be seen from the figure, the simulated results are in good agreement with the experimental values for both the Al_3_Ti and the α-(Al) phases, indicating that the thermodynamic descriptions of the quaternary Al-Si-Mg-Ti system obtained in the present work are reliable.

#### 4.1.2. Quinary Al-Si-Mg-Sr-Ti System and Key Experimental Validation

Based on the reported Al-Si-Mg-Sr system [5] and the updated Al-Si-Mg-Ti system in Section 4.1, the thermodynamic database of the quinary Al-Si-Mg-Sr-Ti system was constructed through direct extrapolation. To justify the reliability of the established thermodynamic database, three key Al-Si-Mg-Sr-Ti alloys (i.e., D1, D2, D3) in the Al-rich corner were prepared. The nominal composition and the actual composition of the three alloys are shown in Table 1. The calculated phase transition temperatures and the solidified microstructure information of the three key alloys based on the established database are comprehensively compared with the experimentally measured data.

**Figure 3:**
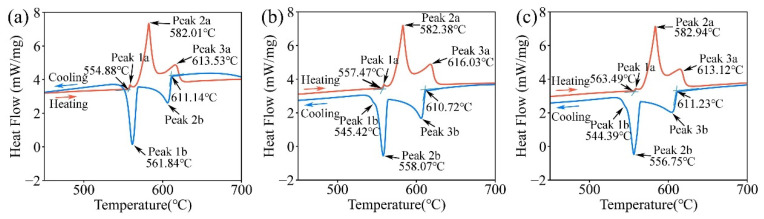
DSC curves during the heating and cooling of Al-Si-Mg-Sr alloys with different Ti contents: (**a**) 0.03 wt.% Ti, (**b**) 0.08 wt.% Ti, and (**c**) 0.2 wt.% Ti. The heating/cooling rate was set to be 10 °C/min.

**Figure 4:**
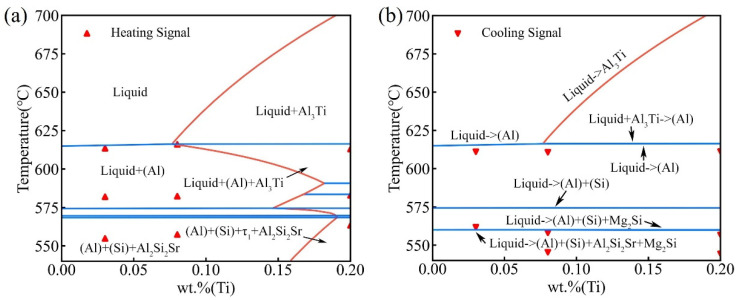
(**a**) Calculated equilibrium vertical section of A356-0.005Sr-*x*Ti (in wt.%), compared with the experimental data from the DSC heating curves; (**b**) constructed solidification diagram of A356-0.005Sr-*x*Ti (in wt.%) using the Scheil-Gulliver mode, compared with the experimental data from the DSC cooling curves.

Figure 3 shows the experimental DSC curves of the three alloys. Three peaks were detected in all three alloys during heating. However, during the cooling process, three peaks were detected in the D2 and D3 alloys, while only two peaks were detected in the D1 alloy. That is because (i) the thermal effect of the peak corresponding to Liquid → (Al) + (Si) + Mg_2_Si/Liquid → (Al) + (Si) + Mg_2_Si + Al_2_Si_2_Sr is too low to be detected with the relatively high cooling rate (i.e., 10 °C/min) [55] and (ii) such a peak is quite close to peak 1b in Figure 3a, and thus, they may overlap with each other under the relatively high cooling rate (i.e., 10 °C/min) [55]. To further analyze the reliability of the thermodynamic database, the phase transition temperatures of the DSC curves in Figure 3 were extracted and compared with the calculated results, as shown in Figure 4.

Figure 4a shows the calculated vertical section of Al-7.0Si-0.4Mg-0.005Sr-*x*Ti based on the presently established thermodynamic database, compared with the experimental heating signals of the D1, D2, and D3 alloys. Three endothermic peaks were observed for each sample during the heating process. For the D1 and D2 alloys, three peaks (from high temperature to low temperature) correspond to the formation of α-(Al), the binary eutectic reaction Liquid → (Al) + (Si), and the ternary eutectic reaction Liquid → (Al) + (Si) + Al_2_Si_2_Sr, respectively. As for the D3 alloy, the first peak and the last peak (corresponding to the respective highest and lowest temperatures) may be the formation of α-Al (Liquid + Al_3_Ti → (Al)/Liquid → (Al)) and the reaction of Liquid → (Al) + (Si) + Al_2_Si_2_Sr + τ_1_, respectively. However, only one peak was detected for several reactions in between. That might be caused by the small temperature interval of those reactions, resulting in the superposition of the peaks. Figure 4b displays the solidification diagram of the Al-7.0Si-0.4Mg-0.005Sr-*x*Ti and the comparison with the cooling signal of the DSC experiment for the D1, D2, and D3 alloys. The three exothermic peaks in D2 and D3 alloys correspond to the reactions: Liquid → (Al) (and D3: Liquid + Al_3_Ti → (Al)), Liquid → (Al) + (Si), and Liquid → (Al) + (Si) + Mg_2_Si, respectively. The two exothermic peaks in the D1 alloy correspond to the reaction: Liquid → (Al) and Liquid → (Al) + (Si), respectively. Regarding the signal of the formation of the Al_3_Ti in D3 alloy that was not detected during the DSC experiment heating/cooling process, the explanation might be that the amount of dispersed Al_3_Ti as heterogeneous nucleation sites of (Al) is low. Based on the present thermodynamic calculations, with an additional Ti content of 0.2 wt.% in A356-0.005Sr alloy, the generated Al_3_Ti content is approximately 0.28%. Moreover, parts of them dissolve in α-(Al). Thus, the thermal effect of the formation of Al_3_Ti should be too low to be detected. What is more, as can be seen from Figure 4b, there is a systematic difference (ca. ±10 °C) between the experimental data from DSC cooling curves and the model-predicted values. That is because the nucleation stage is not taken into account in the Scheil-Gulliver solidification simulation, resulting in a systematic discrepancy between the model predictions and the experimental results.

**Figure 5:**
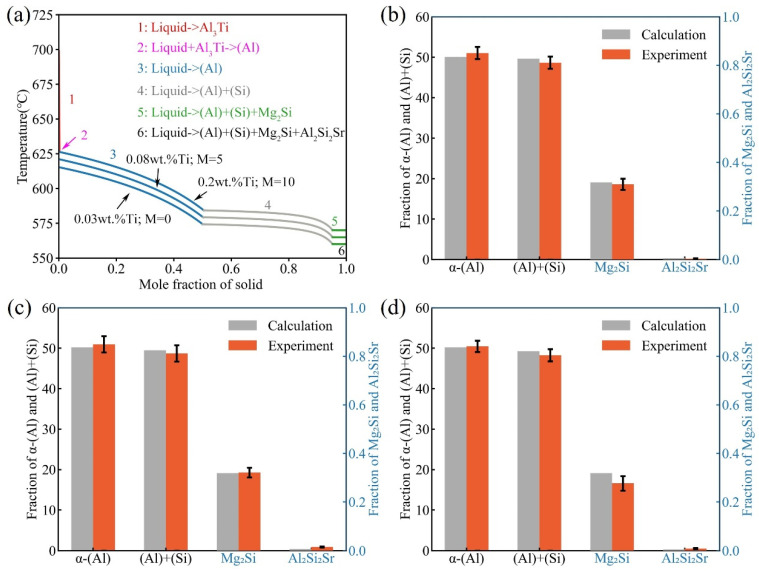
(**a**) Simulated solidification curves of different alloys (Al-7.0Si-0.4Mg-0.005Sr-0.03Ti, Al-7.0Si-0.4Mg-0.005Sr-0.08Ti, and Al-7.0Si-0.4Mg-0.005Sr-0.20Ti) under the Scheil-Gulliver conditions; comparison of the model-predicted volume fractions of α-(Al), (Al)+(Si), Mg_2_Si, and Al_2_Si_2_Sr phase and the measured values in (**b**) Al-7.0Si-0.4Mg-0.005Sr-0.03Ti, (**c**) Al-7.0Si-0.4Mg-0.005Sr-0.08Ti, and (**d**) Al-7.0Si-0.4Mg-0.005Sr-0.20Ti alloys.

To further verify the reliability of the Al-Si-Mg-Sr-Ti quinary thermodynamic database, the experimental solidification microstructures of the three alloys were analyzed and compared with the calculated results. Figure 5 shows the solidification sequences of the three A356-0.005Sr key alloys containing different Ti contents and the comparison between the calculated phase fractions and the experimentally measured results. As shown in Figure 5a, the blue line is the stage of the liquid phase transforming into α-Al, and the yellow and green lines are the stages of liquid transformation into eutectic (Al) and Si (marked as (Al)+(Si)) and liquid transformation into eutectic (Al), eutectic Si, and Mg_2_Si, respectively. In the final stage, the residual liquid phase totally transforms into a quaternary eutectic microstructure consisting of (Al), Si, Mg_2_Si, and Al_2_Si_2_Sr, where Al_2_Si_2_Sr only precipitates in this stage. Additionally, it should be noted that the Al_3_Ti was not found in the experimental solidification microstructures in the present work because of the fact that the amount of the generated Al_3_Ti phase is quite low and it diffusely distributes as heterogeneous nucleation sites with relatively small particles. Figure 5b–d shows the comparison between the model-predicted phase/structure fractions and the experimental measured results for alloys D1, D2, and D3, respectively. As shown in Figure 5b, the model-predicted fractions of the α-(Al), (Al)+(Si), Mg_2_Si, and Al_2_Si_2_Sr in alloy D1 are 50.0489 vol.%, 49.6277 vol.%, 0.3179 vol.%, and 0.0055 vol.%, respectively. These model-predicted values agree very well with the corresponding experimental data (51.04 ± 1.5 vol.%, 48.65 ± 1.5 vol.%, 0.31 ± 0.03 vol.%, and 0.004 ± 0.002 vol.%, respectively) because they locate well within the standard deviation of the experimental data (see the error bars in Figure 5b–d). As shown in Figure 5c,d, the model-predicted phase/structure fractions in the D2 and D3 alloys also match well with the experimental results. It is clearly demonstrated that the phase transition temperature and solidified microstructure information in Figure 4 and Figure 5 validate the reliability of the thermodynamic database for the Al-Si-Mg-Sr-Ti system.

### 4.2. Efficient Design of Optimal Ti in A356-0.005Sr Alloys and Experimental Validation

#### 4.2.1. Alloy Design

**Figure 6:**
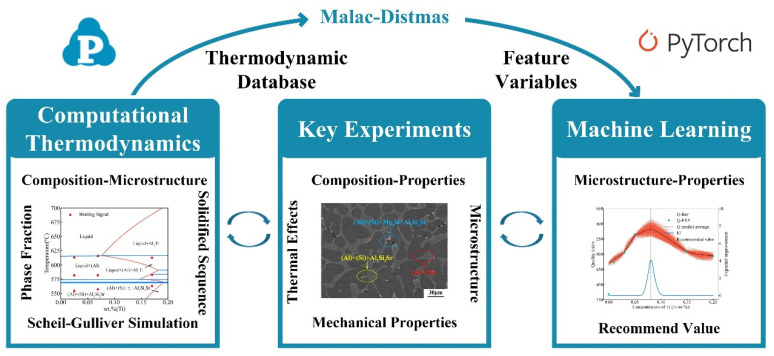
Flow chart of high-efficiency alloy design by integrating computational thermodynamics, machine learning, and key experiments. The thermodynamic database was validated by key experiments and can provide feature variables for machine learning through the computation of the high-throughput platform Malac-Distmas, while the mechanical properties obtained from key experimental tests provide training data for machine learning. After training by machine learning, the points that may have the best comprehensive mechanical properties are recommended by the acquisition function (EI) and validated by key experiments. Finally, combining computational thermodynamics, machine learning, and key experiments establishes the relationship between the “composition-process-microstructure-properties” of alloys to achieve efficient alloy design.

ML can create complex, hidden relationships for these correlated properties as well as the final data. Effective applications of ML for materials design may require a large number of data points and the use of the relevant properties of the material as input. However, generating high-quality data points is time-consuming and expensive, and finding the relevant properties of the materials is more important for the effective application of ML. Fortunately, the CT approach can provide the relevant properties of the materials, such as the solidified microstructure information. Combining a small amount of expensive experimental data, ML is used to establish complex relationships between the inputs and outputs, ultimately providing a relatively reliable prediction for the material. The strategy diagram combining CT and ML methods for alloy designs is shown in Figure 6 and applied to the design of optimal Ti content for A356-0.005Sr.

**Figure 7:**
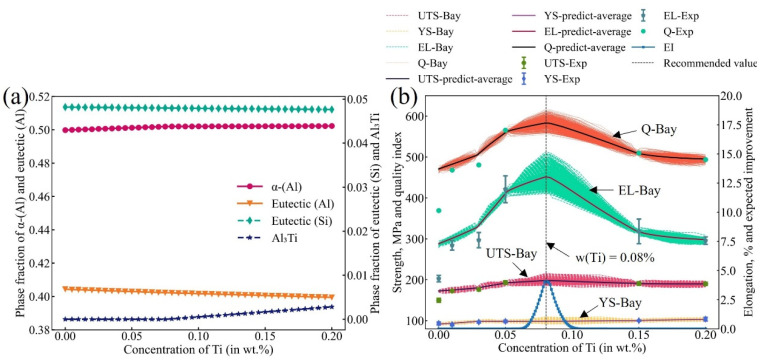
Optimal Ti content design for A356-0.005Sr alloy. (**a**) Fractions of different phases/structures (i.e., a-(Al), eutectic (Al), eutectic Si, and Al_3_Ti) in A356-0.005Sr alloys with different Ti contents obtained by computational thermodynamic; (**b**) simulation results of the mechanical properties, quality index, and the expected improvement of A356-0.005Sr with different Ti content. Solid symbols represent the experimental data or the quality index corresponding to the experimental data. The lower part of the figure represents the expected improvement result based on the quality index, with the recommended point that the Ti content is 0.08 wt.%.

Firstly, the high-throughput simulations of Scheil-Gulliver for a series of A356-0.005Sr alloys with different Ti contents were performed using the Malac-Distmas coupled with Pandat software based on the reliable thermodynamic database of the Al-Si-Mg-Sr-Ti quinary system established in the present work. The fraction for each phase/structure, which is closely related to the mechanical properties of target alloys, was used as the input feature variable of the ML model. In A356-0.005Sr-*x*Ti (*x* = 0–0.2 wt.%) alloys, the α-(Al) and (Al) + (Si), as the main parts of the solidified microstructures, have the greatest influence on the alloy properties. As a heterogeneous nucleation site, Al_3_Ti also has a strong influence on the alloy properties. Moreover, the variation of the Ti content does not affect the contents of Mg_2_Si and Al_2_Si_2_Sr, which were not considered feature variables during ML. Therefore, the phase fractions of α-(Al), eutectic (Al), eutectic Si, and Al_3_Ti, along with different Ti contents, were efficiently retrieved due to the high-throughput simulations of the Scheil-Gulliver solidification, as displayed in Figure 7a. As the Ti content increases over the range of 0–0.2 wt.%, both fractions of the eutectic (Al) and the eutectic (Si) show a slow decrease, while that of the primary (Al) shows a faster increase till 0.078 wt.% Ti, followed by a slow increase. Al_3_Ti starts to precipitate at a Ti content of 0.078 wt.% and gradually increases with a further increase in Ti content. Based on the reliable thermodynamic database, the quantitative relationship of the “composition/processing-microstructure” was established and further densified by ANN I, which can provide composition information with arbitrary accuracy for subsequent point recommendations.

Secondly, several A356-0.005Sr alloys with different Ti contents (0, 0.01, 0.03, 0.05, 0.15, and 0.20 wt.% Ti), i.e., M1–M6 in Table 1, were prepared, and their measured mechanical properties served as the training data for ML. At this stage, the inputs and outputs of the ANN II are the experimental microstructural information and the mechanical properties of alloys (ultimate tensile strength, 0.2% yield strength, and elongation, hereinafter referred to as UTS, YS, and EL, respectively), respectively. After that, the ML within a Bayesian optimization framework was combined with CT to establish the relationship “composition-process-microstructure-properties” in alloys. From the established “composition-process-microstructure-properties” relationship, new data, which take into account the confidence level δ, were inferred from the simulated random compositions, forming an augmented dataset and updating ANN II. This process was performed 1000 times in parallel to obtain the mean value and standard deviation. Based on the simulation results, the recommendation point with the best comprehensive mechanical properties can be obtained by the maximum EI (as shown in Equation(1)). More detail about machine learning can be found in Section S3 of the supplementary materials. Figure S5 shows the distribution of the cumulative distribution function (CDF) and probability density function (PDF) with different Ti contents in A356-0.005Sr in this iteration of Bayesian optimization.

The results of ML are shown in Figure 7b, where the red dashed lines, the yellow dashed lines, and the green dashed lines indicate the predicted UTS, YS, and EL values, respectively, while the corresponding solid lines indicate the mean value of all the simulated processes, respectively. Obviously, the simulated mechanical properties constitute the region of the possible range of mechanical properties of the alloy, i.e., the uncertainty of the predicted mechanical properties. The experimental data were also appended to the figure as the solid symbol. To represent the comprehensive mechanical properties of the alloy more intuitively, the quality index, *Q_DJR_* = *UTS* + 150·log(*EL*) [56], was employed in the present work. The relationship between the quality index and Ti content is given in Figure 7b. In the figure, the orange dashed lines are the ML simulation results, while the black solid line is the average value of the quality index. The quality indices corresponding to the experimental values are represented by solid circles. Finally, the EI of the quality index in the A356-0.005Sr alloy with different Ti contents was calculated and denoted by the blue solid line in Figure 7b. Here, the inputs to the EI are the variance and mean of the uncertainty *Q_DJR_*. As can be clearly seen in Figure 7b, the A356-0.005Sr alloy may exhibit the best mechanical properties at the Ti content of 0.08 wt.%, which represents the recommended optimal alloy composition.

#### 4.2.2. Experimental Validation

**Figure 8:**
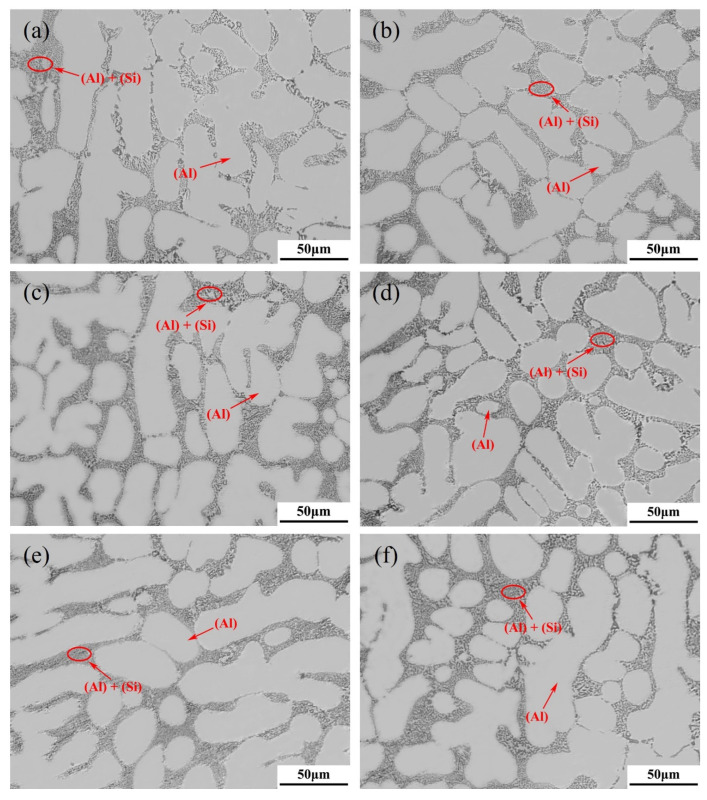
OM images of A356-0.005Sr alloys with different Ti contents: (**a**) 0.01 wt.% Ti; (**b**) 0.03 wt.% Ti; (**c**) 0.05 wt.% Ti; (**d**) 0.08 wt.% Ti; (**e**) 0.15 wt.% Ti; (**f**) 0.20 wt.% Ti.

**Figure 9:**
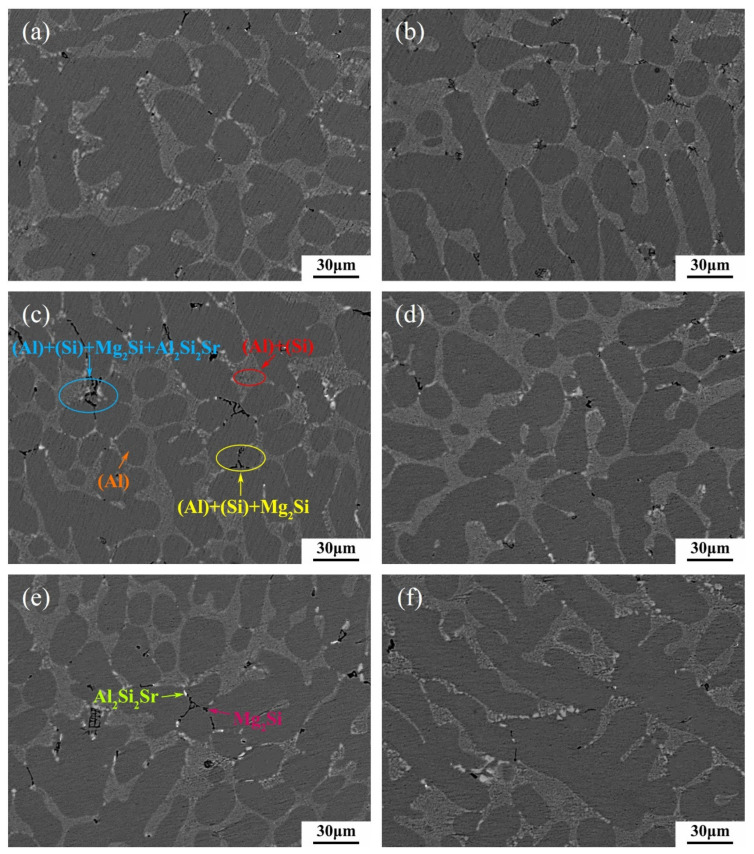
BSE images of A356-0.005Sr alloys with different Ti contents: (**a**) 0.01 wt.% Ti; (**b**) 0.03 wt.% Ti; (**c**) 0.05 wt.% Ti; (**d**) 0.08 wt.% Ti; (**e**) 0.15 wt.% Ti; (**f**) 0.2 wt.% Ti.

In order to verify the optimal Ti content in the alloy A356-0.005Sr recommended by the maximum EI, the alloy A356-0.005Sr-0.08Ti (Al-7.0Si-0.4Mg-0.005Sr-0.08Ti, in wt.%), i.e., O1 in Table 1, was prepared, and its solidified microstructure and mechanical properties were measured. The solidified microstructures of A356-0.005Sr alloys with different Ti contents (including 0.08 wt.%) used for ML in this work are shown in Figure 8 and Figure 9. As indicated in the figures, the solidified microstructures of all the samples consisted of primary α-(Al), eutectic (Al)+(Si), Mg_2_Si, and Al_2_Si_2_Sr, showing no obvious difference in the types of phases/structures. All eutectic (Si) particles have a fine fibrous morphology due to the addition of Sr, which is consistent with the fact previously reported by Yi et al. [5]. As stated in the previous section, the phase of Al_3_Ti was not detected here either. Some information on microstructure has been included as a statistic in Section S4 of the supplementary material. As shown in Figure S6a of the supplementary material, the average grain size of α-(Al) decreases with the addition of Ti. Figure S6c shows the size distribution of α-(Al). At a Ti content of approximately 0.08 wt.% for the A356-0.005Sr alloy, the alloy size distribution is more concentrated in small sizes. It is consistent with the average grain size of α-(Al) and the mechanical properties of the alloy. A more detailed analysis of the microstructure can be seen in Section S4 of the supplementary material.

**Figure 10:**
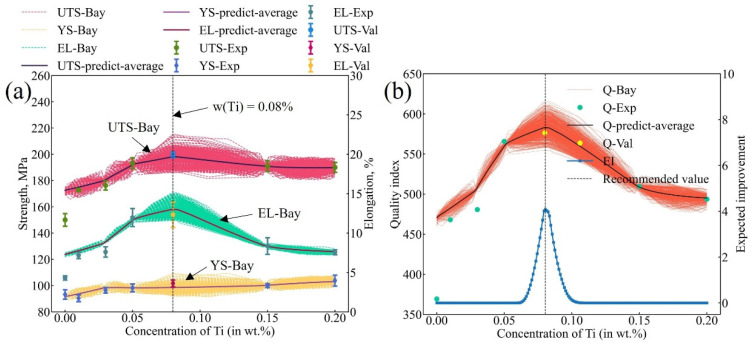
Experimental verification results of predicted optimal Ti content in A356-0.005Sr alloy. (**a**) Experimental results of mechanical properties with predicted optimal Ti content in A356-0.005Sr alloy (i.e., 0.08%, the corresponding results are represented as UTS-Val, YS-Val, and EL-Val). (**b**) Experimental validation results expressed as a quality index, where the quality index for optimal Ti content is represented as Q-Val.

The measured mechanical properties of the A356-0.005Sr-0.08Ti alloy are displayed in Figure 10a, i.e, that the ultimate tensile strength (UTS), 0.2% yield strength (YS), and elongation (EL) are 199.6 MPa, 101.6 MPa, and 12.3%, respectively. Obviously, the experimental values agree well with the predicted ones. At the designed composition, both UTS and EL reached the highest values, while the YS also kept a relatively high value. Figure 10b shows the validation results in terms of the quality index. Again, the experimental quality index of the A356-0.005Sr-0.08Ti alloy represented the highest value, and also fits well with the predicted data. To further prove the reliability of the optimal additional Ti content in the A356-0.005 Sr alloy designed in this work, the experimental data of the A356-0.005Sr-0.08Ti alloy were fed back to the dataset for the next iteration of the Bayesian optimization. The relevant proofs are presented in Section S5 of the supplementary material. Figure S7 shows the distribution of the cumulative distribution function (CDF) and probability density function (PDF) with different Ti content in A356-0.005Sr in the second iteration of Bayesian optimization. Figure S8 shows machine learning results after adding the new experimental data in the A356-0.005Sr alloy with different Ti contents. The results of the updated ML indicate that the next recommendation is almost identical to the optimal result. It indicates that the Bayesian optimization trend stabilizes. From the above analysis, it can be concluded that the alloy design results based on the Bayesian optimization framework are reliable.

This work established the relationship between the Ti contents and the microstructures of the A356-0.005Sr alloy using phase diagrams and high-throughput calculations based on the CALPHAD technique; the relationship between microstructure and properties was established by machine learning. The optimal Ti content of 0.08 wt.% in the A356-0.005Sr alloy was finally designed, which is consistent with the fact that the common Ti content in industrial production is below 0.15 wt.% and the optimal composition of Ti in the Al-7Si alloy should be a low content as reported by Chen [19] and Li [20]. This reinforces the reasonableness of the present work. Moreover, for the composition design of high-dimensional alloys, the conventional trial-and-error method may cause expensive experimental costs, while the combination of CALPHAD and Bayesian optimization can provide the potential to design high-dimensional composition alloys at low costs.

## 5. Conclusions

- All boundaries binary/ternary systems were first unified, and the thermodynamic databases of Al-Si-Mg-Ti and Al-Si-Mg-Sr-Ti systems were then directly extrapolated from the boundaries. Their reliability was validated by the experimental data from the literature and the present work.
- Combining CT, key experiments, and ML within the Bayesian optimization framework, the quantitative relationship “composition/processing-microstructure-properties” of A356-0.005Sr with different Ti contents was constructed. Based on the evaluated acquisition function EI values, the A356-0.005Sr alloy with an additional 0.08 wt.% Ti was designed to own the best performance point (UTS = 199.6 MPa, YS = 101.6 MPa, and EL = 12.3%) and was finally experimentally validated.
- The successful design of Ti-modified A356-Sr alloys indicated that combining ML, CT, and key experiments within the Bayesian optimization framework is one of the most efficient alloy design methods when there is a small experimental dataset. Meanwhile, with the acquisition function EI, the optimal alloy composition with the best comprehensive properties can be directly recommended, resulting in the avoidance of blindly conducting expensive experiments and human involvement in the next iteration. This can greatly reduce the difficulty of sampling in the complex composition space. Therefore, the presently proposed integration method is anticipated to serve as a general one for alloy design, especially the design of alloys with high-dimensional composition space.

## Figures and Tables

**Table 1:** List of nominal and actual composition of prepared Al-Si-Mg-Sr-Ti alloys in this work.

No. *	Nominal Compositions (wt.%)	Actual Compositions Measured by ICP and CA (wt.%) **				
		Al	Si	Mg	Sr	Ti
D1	Al-7.0Si-0.45Mg-0.005Sr-0.03Ti	Bal.	6.63	0.42	0.0036	0.025
D2	Al-7.0Si-0.45Mg-0.005Sr-0.08Ti	Bal.	6.96	0.41	0.0043	0.069
D3	Al-7.0Si-0.45Mg-0.005Sr-0.20Ti	Bal.	7.08	0.39	0.0031	0.170
M1	Al-7.0Si-0.45Mg-0.005Sr	Bal.	6.52	0.38	0.0041	/
M2	Al-7.0Si-0.45Mg-0.005Sr-0.01Ti	Bal.	7.08	0.33	0.0041	0.010
M3	Al-7.0Si-0.45Mg-0.005Sr-0.03Ti	Bal.	6.63	0.36	0.0032	0.024
M4	Al-7.0Si-0.45Mg-0.005Sr-0.05Ti	Bal.	6.86	0.35	0.0033	0.037
M5	Al-7.0Si-0.45Mg-0.005Sr-0.15Ti	Bal.	7.21	0.37	0.0027	0.11
M6	Al-7.0Si-0.45Mg-0.005Sr-0.20Ti	Bal.	7.08	0.37	0.0028	0.15
O1	Al-7.0Si-0.45Mg-0.005Sr-0.08Ti	Bal.	6.96	0.36	0.0037	0.057

* Alloys of D series for thermodynamic database validation, alloys of M series for machine learning, and alloys of O series for validation of the optimal composition. ** Elements Sr, Mg, and Ti were measured by ICP, while element Si was measured by the CA method.

## Data Availability

The relevant data and scripts used in this study are available upon reasonable request from the corresponding author.

## Supplementary Materials

The following supporting information can be downloaded at: https://www.mdpi.com/article/10.3390/ma16010306/s1, Figure S1: The Al-Si-Ti isothermal sections calculated by the latest database compared with experimental data [20] at different temperatures. (a) 550 °C, (b) 650 °C, (c) 727 °C; Figure S2: The Al-Si-Ti vertical sections were calculated by the latest database, and the experimental data came from Chen [19] and Dezellus [57] respectively. (a) Al-9.0 at. % Si, (b) Al-13.0 at. % Si, (c) Al-18 at. % Si; Figure S3: The liquidus projection of Al-Si-Ti. The experimental data were obtained from Dezellus [57] and Peronnet [58] respectively. The diamond and triangle symbols represent the liquid compositions in equilibrium with Al_3_Ti and τ_1_ (τ_2_), respectively. Red and green symbols represent the measured and interpolated or extrapolated compositions, respectively [19]; Figure S4: Schematic diagram of the process of establishing the thermodynamic database of Al-Si-Mg-Sr-Ti quinary system; Figure S5: Distribution of the cumulative distribution function (CDF) (a) and probability density function (PDF) (b) with x. The x is equal to the Ti content in A356-0.005Sr; Figure S6: Analysis of strengthening and toughing mechanisms by experiments and computational thermodynamic. (a) the average grain size of α-(Al) in A356-0.005Sr-xTi alloys; (b) calculated growth restriction factor Q_true_ with different Ti content for A356-0.005Sr, (c) the size distribution of α-(Al) in A356 alloys with different Ti additional contents; Figure S7: Distribution of the cumulative distribution function (CDF) (a) and probability density function (PDF) (b) with x. The x is equal to the Ti content in A356-0.005Sr in the second iteration of Bayesian optimization; Figure S8: Machine learning results after adding the new experimental data in the A356-0.005Sr alloy with different Ti contents. (a) Simulation results of mechanical properties after adding the experimental values of the recommended point by EI, the solid symbols represent the experimental data (including the recommended point), (b) Quality index, and expected improvement value of A356-0.005Sr with different Ti content computed from the simulation results and experimental data, the black dotted line represents next recommended value by EI. References [59,60,61,62,63,64,65,66,67,68,69,70,71,72] are cited in the supplementary materials.
