# Supplementary material for: Computer Alloy Design of Ti Modified Al-Si-Mg-Sr Casting Alloys for Achieving Simultaneous Enhancement in Strength and Ductility
Source: Materials (Basel). 2022 Dec 28;16(1):306. doi: 10.3390/ma16010306 (PMC9822033; doi:10.3390/ma16010306)
Supplement: Supplementary file 1 [file materials-16-00306-s001.zip › materials-2060531-supplementary.pdf]

Supplementary material

Computer Alloy Design of Ti Modified Al-Si-Mg-Sr Casting Alloys for Achieving Simultaneous Enhancement in Strength and Ductility

Shaoji Zhang [1], Wang Yi [1], Jing Zhong [1], Jianbao Gao [1,*], Zhao Lu [2] and Lijun Zhang [1,*]

1 State Key Laboratory of Powder Metallurgy, Central South University, Changsha 410083, China
2 School of Materials Science and Engineering, Guangxi Key Laboratory of Information Materials, Guilin University of Electronic Technology, Guilin 510004, China
Correspondence: jianbao.gao@csu.edu.cn (J.G.); lijun.zhang@csu.edu.cn (L.Z.)

1. The verification of the Al-Si-Ti ternary system database

The latest thermodynamic descriptions of Al-Si-Ti ternary system were obtained after the unification of the boundary binary system. Figure S1 shows the Al-Si-Ti isothermal sections at three different temperatures calculated from the latest thermodynamic database. From the figure, the isothermal sections do not differ significantly from the original literature [1] and are in good agreement with the experimental data [1]. Figure S2 shows three different vertical sections of the Al-Si-Ti ternary system calculated at the Al-rich corner using the latest database. The form of the graph in Figure S2 is from Li [1], and the experimental data are from Chen [2] and Dezellus [3] respectively. The vertical sections calculated by the latest thermodynamic description differ slightly from the original literature but are in good agreement with the experimental data as well. Figure S3 shows the Al-Si-Ti liquidus projection below 900℃ calculated from the original Al-Si-Ti ternary system form [1] using the latest database. It is generally agreed with the experimental data.

By comparing calculated results with the experimental data, the Al-Si-Ti thermodynamic database after unifying the boundary system is reliable.

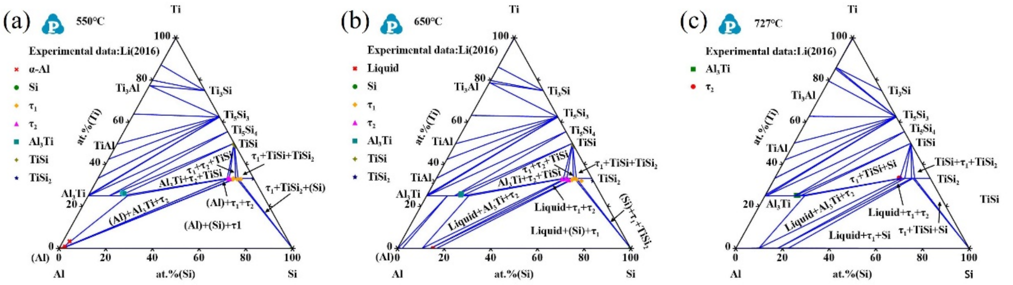

Figure S1. The Al-Si-Ti isothermal sections calculated by the latest database compared with experimental data [1] at different temperatures. (a) 550℃, (b) 650℃, (c) 727℃.

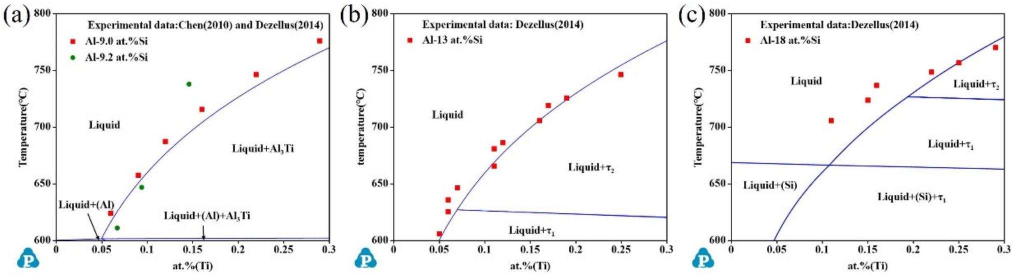

Figure S2. The Al-Si-Ti vertical sections were calculated by the latest database, and the experimental data came from Chen [2] and Dezellus [3] respectively. (a) Al-9.0 at. % Si, (b) Al-13.0 at. % Si, (c) Al-18 at. % Si.

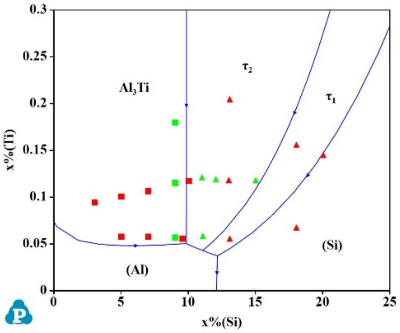

Figure S3. The liquidus projection of Al-Si-Ti. The experimental data were obtained from Dezellus [3] and Peronnet [4] respectively. The diamond and triangle symbols represent the liquid compositions in equilibrium with Al_3Ti and τ_1 (τ_2), respectively. Red and green symbols represent the measured and interpolated or extrapolated compositions, respectively [1].

2. Thermodynamic models

There are 10 binary systems and 10 ternary systems in the Al-Si-Mg-Sr-Ti quinary system. The Al-Si [6], Al-Mg [6], Al-Sr [7], Al-Ti [8], Mg-Si [6], Mg-Sr [9], Si-Sr [10], Si-Ti [1], Sr-Ti [11] binary systems were directly adopted based on the critical review of the literature data by CALPHAD approach. As for Mg-Ti binary system, Murry [12] carried out the Mg-Ti system in 1986. In the Mg-Ti binary equilibrium phase diagram, there are no binary phases and even the two elements are totally immiscible in liquid form [13]. Therefore, thermodynamic descriptions of the Mg-Ti system were directly extrapolated from two pure elements in this work.

Besides, the literature data on Al-Si-Mg [6], Al-Si-Sr [10], Al-Mg-Sr [14], and Al-Si-Ti [1] ternary systems were employed in this work. It should be noted that the sub-binary Al-Si of Al-Si-Ti ternary and Al-Si-Mg ternary system is inconsistent, and the Al-Si sub-binary system of Al-Si-Mg system is adopted in this paper, the database compatibility should be verified. The Al-Mg-Ti system has been less studied. In 1987, Kerimov et al [15] reported that there is a ternary compound $Al_{18}Ti_2Mg_3$ in Al-Mg-Ti ternary system. Subsequently, $Al_{18}Ti_2Mg_3$ has been observed and studied by many researchers [16–18], but no thermodynamic description of the Al-Mg-Ti ternary system has been established. Considering that the main task in this study is the material design for Al-Si-(Mg) series alloy and the thermodynamic descriptions of the Al-rich region, the Al-Mg-Ti ternary system is not considered. Therefore, the Al-Mg-Ti ternary system is established by extrapolating from boundary binary systems. Similarly, the Al-Sr-Ti, Si-Mg-Sr, Si-Mg-Ti, Si-Sr-Ti, and Mg-Sr-Ti ternary systems are no longer discussed in this work. They are directly obtained by extrapolation from corresponding boundary binary systems.

One of the important purposes of this work is to study the optimum amount of Sr and Ti in Al-Si-(Mg) alloy, there are two major quaternary systems in the quinary system: Al-Si-Mg-Sr and Al-Si-Mg-Ti quaternary systems. The Al-Si-Mg-Sr quaternary system has been established and verified by our group [19], so it is directly utilized in the present work. However, there is no reliable thermodynamic data for the Al-Si-Mg-Ti quaternary system, at the same time, there is no literature report that the Al-Si-Mg-Ti system contains quaternary compounds. Therefore, the thermodynamic descriptions of the Al-Si-Mg-Ti quaternary system were obtained by extrapolation from the ternary system and verified by experimental data. Finally, the Al-Si-Mg-Sr-Ti quinary system was obtained by extrapolating from Al-Si-Mg-Sr and Al-Si-Mg-Ti quaternary systems.

A schematic diagram of the process of establishing the thermodynamic database of the Al-Si-Mg-Sr-Ti quinary system is shown in Figure S4. Where green indicates direct adoption of literature data, black indicates no relevant data in the literature or no solid solution gap between two elements in the binary system and no binary compound. Blue indicates that the boundary system has been updated by unification in this work and needs to be revalidated. Red indicates that the thermodynamic databse was obtained by extrapolation and needs to be validated by experimental data from the literature or this work. And all thermodynamic parameters of the pure element were obtained from Dinsdale [5].

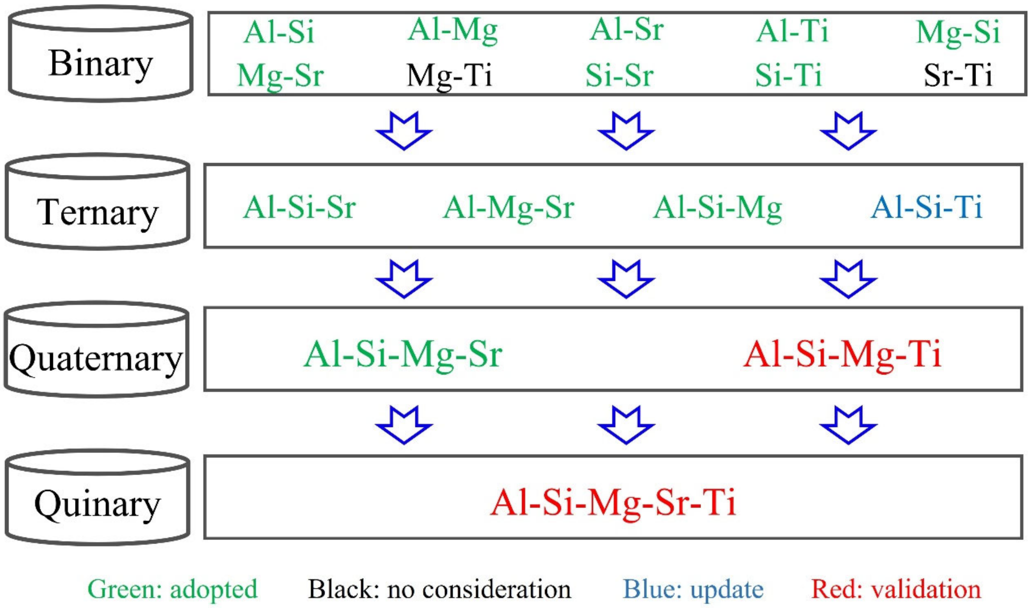

Figure S4. Schematic diagram of the process of establishing the thermodynamic database of Al-Si-Mg-Sr-Ti quinary system.

In the following, the analytical expressions for the Gibbs energies of part involved phases are briefly presented.

2.1. Pure elements

The Gibbs energy function for the pure element i (i = Al, Si, Mg, Sr, or Ti) in the unary phase can be expressed as [20]:

$$G_i(\mathrm{T}) - \mathrm{H}_i^{\mathrm{SER}} = A + BT + CT\ln T + DT^2 + ET^{-1} + FT^3 + IT^{-7} + JT^{-9} \quad \text{(S1)}$$

where H_i^{SER} is the molar enthalpy of the element i at 298.15 k, 1 bar in its standard element reference (SER) state, and T is the absolute temperature. The term IT^{-7} is used for a liquid below the melting point and JT^{-9} is used for a solid phase above the melting point. The thermodynamic parameters for the pure elements were obtained from the compilation of Dinsdale [20].

2.2. Solution phases

In the Al-Si-Mg-Sr-Ti quinary system, liquid, fcc, bcc, hcp, and (Si) solution phases are described by the substitutional solution model. Considering the liquid phase, for example, the molar Gibbs energy is expressed by the following equations [21,22]:

$$G^{\mathrm{liquid}} = {}^{\mathrm{ref}}G^{\mathrm{liquid}} + {}^{\mathrm{mix}}G^{\mathrm{liquid}} + {}^{\mathrm{ex}}G^{\mathrm{liquid}}_{\mathrm{bin}} + {}^{\mathrm{ex}}G^{\mathrm{liquid}}_{\mathrm{ter}} + {}^{\mathrm{ex}}G^{\mathrm{liquid}}_{\mathrm{qua}} + {}^{\mathrm{ex}}G^{\mathrm{liquid}}_{\mathrm{qui}} \quad \text{(S2)}$$

with

$${}^{\mathrm{ref}}G^{\mathrm{liquid}} = x_{\mathrm{Al}}\,{}^{0}G^{\mathrm{liquid}}_{\mathrm{Al}} + x_{\mathrm{Si}}\,{}^{0}G^{\mathrm{liquid}}_{\mathrm{Si}} + x_{\mathrm{Mg}}\,{}^{0}G^{\mathrm{liquid}}_{\mathrm{Mg}} + x_{\mathrm{Sr}}\,{}^{0}G^{\mathrm{liquid}}_{\mathrm{Sr}} + x_{\mathrm{Ti}}\,{}^{0}G^{\mathrm{liquid}}_{\mathrm{Ti}} \quad \text{(S3)}$$

$${}^{\mathrm{mix}}G^{\mathrm{liquid}} = RT(x_{\mathrm{Al}}\ln x_{\mathrm{Al}} + x_{\mathrm{Si}}\ln x_{\mathrm{Si}} + x_{\mathrm{Mg}}\ln x_{\mathrm{Mg}} + x_{\mathrm{Sr}}\ln x_{\mathrm{Sr}} + x_{\mathrm{Ti}}\ln x_{\mathrm{Ti}}) \quad \text{(S4)}$$

$$
\begin{aligned}
{}^{ex}G^{liquid}_{bin} =& x_{Al}x_{Si}\sum_{i=0}^{n}(x_{Al}-x_{Si})^i L^i_{AlSi} + x_{Al}x_{Mg}\sum_{i=0}^{n}(x_{Al}-x_{Mg})^i L^i_{AlMg} \\
&+ x_{Al}x_{Sr}\sum_{i=0}^{n}(x_{Al}-x_{Sr})^i L^i_{AlSr} + x_{Al}x_{Ti}\sum_{i=0}^{n}(x_{Al}-x_{Ti})^i L^i_{AlTi} \\
&+ x_{Si}x_{Mg}\sum_{i=0}^{n}(x_{Si}-x_{Mg})^i L^i_{SiMg} + x_{Si}x_{Sr}\sum_{i=0}^{n}(x_{Si}-x_{Sr})^i L^i_{SiSr} \\
&+ x_{Si}x_{Ti}\sum_{i=0}^{n}(x_{Si}-x_{Ti})^i L^i_{SiTi} + x_{Mg}x_{Sr}\sum_{i=0}^{n}(x_{Mg}-x_{Sr})^i L^i_{MgSr} \\
&+ x_{Mg}x_{Ti}\sum_{i=0}^{n}(x_{Mg}-x_{Ti})^i L^i_{MgTi} + x_{Sr}x_{Ti}\sum_{i=0}^{n}(x_{Sr}-x_{Ti})^i L^i_{SrTi}
\end{aligned}
\quad (S5)
$$

$$
\begin{aligned}
{}^{ex}G^{liquid}_{ter} =& x_{Al}x_{Si}x_{Mg}\sum_{j=Al,Si,Mg}^{n}[x_j + (1-x_{Al}-x_{Si}-x_{Mg})/3]\cdot L^j_{AlSiMg} \\
&+ x_{Al}x_{Si}x_{Sr}\sum_{j=Al,Si,Sr}^{n}[x_j + (1-x_{Al}-x_{Si}-x_{Sr})/3]\cdot L^j_{AlSiSr} \\
&+ x_{Al}x_{Si}x_{Ti}\sum_{j=Al,Si,Ti}^{n}[x_j + (1-x_{Al}-x_{Si}-x_{Ti})/3]\cdot L^j_{AlSiTi} \\
&+ x_{Al}x_{Mg}x_{Sr}\sum_{j=Al,Mg,Sr}^{n}[x_j + (1-x_{Al}-x_{Mg}-x_{Sr})/3]\cdot L^j_{AlMgSr} \\
&+ x_{Al}x_{Mg}x_{Ti}\sum_{j=Al,Mg,Ti}^{n}[x_j + (1-x_{Al}-x_{Mg}-x_{Ti})/3]\cdot L^j_{AlMgTi} \\
&+ x_{Al}x_{Sr}x_{Ti}\sum_{j=Al,Sr,Ti}^{n}[x_j + (1-x_{Al}-x_{Sr}-x_{Ti})/3]\cdot L^j_{AlSrTi} \\
&+ x_{Si}x_{Mg}x_{Sr}\sum_{j=Si,Mg,Sr}^{n}[x_j + (1-x_{Si}-x_{Mg}-x_{Sr})/3]\cdot L^j_{SiMgSr} \\
&+ x_{Si}x_{Mg}x_{Ti}\sum_{j=Si,Mg,Ti}^{n}[x_j + (1-x_{Si}-x_{Mg}-x_{Ti})/3]\cdot L^j_{SiMgTi} \\
&+ x_{Si}x_{Sr}x_{Ti}\sum_{j=Si,Sr,Ti}^{n}[x_j + (1-x_{Si}-x_{Sr}-x_{Ti})/3]\cdot L^j_{SiSrTi} \\
&+ x_{Mg}x_{Sr}x_{Ti}\sum_{j=Mg,Sr,Ti}^{n}[x_j + (1-x_{Mg}-x_{Sr}-x_{Ti})/3]\cdot L^j_{MgSrTi}
\end{aligned}
\quad (S6)
$$

$$
\begin{aligned}
{}^{ex}G^{liquid}_{qua} =& x_{Al}x_{Si}x_{Mg}x_{Sr}L_{AlSiMgSr} + x_{Al}x_{Si}x_{Mg}x_{Ti}L_{AlSiMgTi} + x_{Al}x_{Si}x_{Sr}x_{Ti}L_{AlSiSrTi} \\
&+ x_{Al}x_{Mg}x_{Sr}x_{Ti}L_{AlMgSrTi} + x_{Si}x_{Mg}x_{Sr}x_{Ti}L_{SiMgSrTi}
\end{aligned}
\quad (S7)
$$

$$
{}^{ex}G^{liquid}_{qui} = x_{Al}x_{Si}x_{Mg}x_{Sr}x_{Ti}L_{AlSiMgSrTi} \quad (S8)
$$

Where x_i (i=Al, Si, Mg, Sr, or Ti) is the mole fraction of element i, R is the gas content, and T is the temperature in Kelvin. ${}^{0}G^{liquid}_{i}$ (i = Al, Si, Mg, Sr, or Ti) is the molar Gibbs energy of the pure element i in the liquid state. L^{i}_{AB} (A, B = Al, Si, Mg, Sr or Ti, and A ≠ B), L^{i}_{ABC} (A, B, C = Al, Si, Mg, Sr or Ti, and A ≠ B ≠ C), L^{i}_{ABCD} (A, B, C, D = Al, Si, Mg, Sr or Ti, and A ≠ B ≠ C ≠ D) and $L_{AlSiMgSrTi}$ are the binary, ternary, quaternary and quinary interaction parameters, respectively. All these parameters are temperature-dependent and usually expressed as a + b · T. The interaction coefficients, a, and b, are either optimized based on the experimental data or computed from first-principles calculations [23].

2.3. Intermetallic compounds

Regarding intermetallic compounds, this work takes Al-Si-Ti ternary system as an example to illustrate. In the Al-Si-Ti ternary system, there are 10 boundary binary systems, including AlTi, Al_5Ti_2, $AlTi_3$, Al_2Ti, Al_3Ti, Ti_3Si, Ti_5Si_3, Ti_5Si_4, TiSi, $TiSi_2$. No ternary solubility was experimentally observed in the Al_5Ti_2, Al_2Ti, Ti_3Si, Ti_5Si_3, Ti_5Si_4, TiSi, $TiSi_2$, so these compounds are considered pure binary compounds and they are described as stoichiometric model. For example, the Gibbs energy of Al_2Ti per mole atom is given as:

$$
G^{Al_2Ti} = 0.33333\,{}^{0}G^{fcc}_{Al} + 0.66667\,{}^{0}G^{fcc}_{Ti} + a + bT \quad (S9)
$$

in which ${}^{0}G^{fcc}_{Al}$ and ${}^{0}G^{fcc}_{Ti}$ are the Gibbs energy of pure Al and Ti in their stable states, respectively. The coefficients a and b are contents.

As for AlTi, $AlTi_3$, and Al_3Ti, there were experimentally observed ternary solubility. So AlTi, $AlTi_3$, and Al_3Ti were described by sublattice model. Thus they were modeled as $(Ti, Al)_{0.5}$ $(Ti, Al)_{0.5}$, $(Ti, Al)_{0.25}$ $(Ti, Al)_{0.75,}$ and $(Ti, Si, Al)_{0.75}$ $(Ti, Al)_{0.25}$ respectively. For the Al_3Ti phase, there was a solubility limit of Si, and the Si atom was confirmed to substitute the Al position according to Li [1]. Following Li [1], the Al_3Ti phase is thus described by the sub-lattice model $(Al, Si)_3Ti$. According to the formula for the sub-lattice model [24], the Gibbs energy of Al_3Ti per mole atom can be expressed as:

$$\begin{aligned}
&G^{\mathrm{Al_3Ti}} = y'_{\mathrm{Al}}y''_{\mathrm{Ti}}G^{\mathrm{Al_3Ti}}_{\mathrm{Al:Ti}} + y'_{\mathrm{Al}}y''_{\mathrm{Al}}G^{\mathrm{Al_3Ti}}_{\mathrm{Al:Al}} + y'_{\mathrm{Si}}y''_{\mathrm{Ti}}G^{\mathrm{Al_3Ti}}_{\mathrm{Si:Ti}} + y'_{\mathrm{Si}}y''_{\mathrm{Al}}G^{\mathrm{Al_3Ti}}_{\mathrm{Si:Al}} \\
&+y'_{\mathrm{Ti}}y''_{\mathrm{Al}}G^{\mathrm{Al_3Ti}}_{\mathrm{Ti:Al}} + y'_{\mathrm{Ti}}y''_{\mathrm{Ti}}G^{\mathrm{Al_3Ti}}_{\mathrm{Ti:Ti}} \\
&+0.75RT(y'_{\mathrm{Al}}\ln y'_{\mathrm{Al}} + y'_{\mathrm{Si}}\ln y'_{\mathrm{Si}} + y'_{\mathrm{Ti}}\ln y'_{\mathrm{Ti}}) \\
&+0.25RT(y''_{\mathrm{Al}}\ln y''_{\mathrm{Al}} + y''_{\mathrm{Ti}}\ln y''_{\mathrm{Ti}}) \qquad \text{(S10)} \\
&+y'_{\mathrm{Al}}y'_{\mathrm{Ti}}y''_{\mathrm{Ti}}\,{}^{0}L^{\mathrm{Al_3Ti}}_{\mathrm{Al,Ti:Ti}} + y'_{\mathrm{Al}}y'_{\mathrm{Ti}}y''_{\mathrm{Al}}\,{}^{0}L^{\mathrm{Al_3Ti}}_{\mathrm{Al,Ti:Al}} + y'_{\mathrm{Al}}y''_{\mathrm{Ti}}y''_{\mathrm{Al}}\,{}^{0}L^{\mathrm{Al_3Ti}}_{\mathrm{Al:Al,Ti}} + y'_{\mathrm{Ti}}y''_{\mathrm{Al}}y''_{\mathrm{Ti}}\,{}^{0}L^{\mathrm{Al_3Ti}}_{\mathrm{Ti:Al,Ti}} \\
&+y'_{\mathrm{Al}}y'_{\mathrm{Si}}y''_{\mathrm{Ti}}[{}^{0}L^{\mathrm{Al_3Ti}}_{\mathrm{Al,Si:Ti}} + {}^{1}L^{\mathrm{Al_3Ti}}_{\mathrm{Al,Si:Ti}}(y'_{\mathrm{Al}} - y'_{\mathrm{Si}})]
\end{aligned}$$

where y'_{Al} and y'_{Si} are the site fractions of Al and Si on the first sub-lattice, respectively. The end-member energy $G^{\mathrm{Al_3Ti}}_{\mathrm{Al:Ti}}$ represents the Gibbs energy of the stable binary Al_3Ti phase and was obtained from the description of the Al-Ti data set [8]. The parameter denoted as $G^{\mathrm{Al_3Ti}}_{\mathrm{Si:Ti}}$ represents the Gibbs energy of the fictitious binary Si_3Ti compound. A sufficiently large and positive value is given to $G^{\mathrm{Al_3Ti}}_{\mathrm{Si:Ti}}$ reflect the instability of this hypothetical end-member. $L^{\mathrm{Al_3Ti}}_{\mathrm{Al,Si:Ti}}$ is the regular ternary parameter that is evaluated from the experimental data.

In the Al-Si-Ti ternary system, τ_1 and τ_2 are in the form of ternary stoichiometric compounds. They were both modeled as three sublattice models $(Ti)_{0.33333}(Al, Si)_{0.333333}(Al, Si)_{0.33333}$, which means that Ti occupied one type of lattice site alone and Al and Si occupied the other types of lattice sites. The corresponding Gibbs energy per mole atoms for both τ_1 and τ_2 is expressed as [1]:

$$\begin{aligned}
&G^{\tau} = y''_{\mathrm{Al}}y'''_{\mathrm{Al}}G^{\tau}_{\mathrm{Ti:Al:Al}} + y''_{\mathrm{Al}}y'''_{\mathrm{Si}}G^{\tau}_{\mathrm{Ti:Al:Si}} + y''_{\mathrm{Si}}y'''_{\mathrm{Al}}G^{\tau}_{\mathrm{Ti:Si:Al}} + y''_{\mathrm{Si}}y'''_{\mathrm{Si}}G^{\tau}_{\mathrm{Ti:Si:Si}} \\
&+0.33333RT(y''_{\mathrm{Al}}\ln y''_{\mathrm{Al}} + y''_{\mathrm{Si}}\ln y''_{\mathrm{Si}}) + 0.33333RT(y'''_{\mathrm{Al}}\ln y'''_{\mathrm{Al}} + y'''_{\mathrm{Si}}\ln y'''_{\mathrm{Si}}) \qquad \text{(S11)} \\
&+y''_{\mathrm{Al}}y''_{\mathrm{Si}}y'''_{\mathrm{Al}}\,{}^{0}L^{\tau}_{\mathrm{Ti:Al,Si:Al}} + y''_{\mathrm{Al}}y''_{\mathrm{Si}}y'''_{\mathrm{Si}}\,{}^{0}L^{\tau}_{\mathrm{Ti:Al,Si:Si}} + y''_{\mathrm{Al}}y'''_{\mathrm{Al}}y'''_{\mathrm{Si}}\,{}^{0}L^{\tau}_{\mathrm{Ti:Al:Al,Si}} \\
&+y''_{\mathrm{Si}}y'''_{\mathrm{Al}}y'''_{\mathrm{Si}}\,{}^{0}L^{\tau}_{\mathrm{Ti:Si:Al,Si}}
\end{aligned}$$

in which y''_{Al} and y''_{Si} are site fractions of Al and Si on the second sublattice, and, y'''_{Al} and y'''_{Si} are for the third sublattice. The terms of ${}^{0}L^{\tau}_{\mathrm{Ti:Al,Si:Al}}$, ${}^{0}L^{\tau}_{\mathrm{Ti:Al,Si:Si}}$, ${}^{0}L^{\tau}_{\mathrm{Ti:Al:Al,Si}}$, and ${}^{0}L^{\tau}_{\mathrm{Ti:Si:Al,Si}}$ represent the interaction parameters that are functions of temperature. It is assumed that the interaction of Al and Si on the second and the third sublattices is similar, so the values of ${}^{0}L^{\tau}_{\mathrm{Ti:Al,Si:Al}}$, ${}^{0}L^{\tau}_{\mathrm{Ti:Al,Si:Si}}$, ${}^{0}L^{\tau}_{\mathrm{Ti:Al:Al,Si}}$, and ${}^{0}L^{\tau}_{\mathrm{Ti:Si:Al,Si}}$ were set to be the same.

3. Details of machine learning

In the first iteration, the structures of ANN I (1x2x4, input feature is the Ti content, one hidden layer with 8 nodes and another with 7 nodes, output features are solidified microstructures, including the fraction of primary (Al), eutectic (Al), eutectic (Si), and Al_3Ti phase) and ANN II (4x2x3, input features are solidified microstructures as mentioned before, both hidden layers with 9 nodes, output features are mechanical properties, including UTS, YS, and EL) were obtained in this work, but it worth noting that the model of ANN II has been updated in the subsequent 1000 simulations. In the second iteration, the structures of ANN I were directly adopted in the first iteration. The structure of ANN 2 is 4x2x3, where the input and output layers are the same as the first iteration, solidified microstructures and mechanical properties respectively, and the number of nodes in the two hidden layers are 9 and 10 respectively. The computation processes of the first iteration are carried on CPU: Intel(R) Xeon (R) CPU E5-2660 v3@2.60 GHz. For ANN I and ANN II, the consumed time for convergence is both less than 1 hour. As for the Bayesian optimization processes, the average consumed time is about 18 hours when 1000 computation simulations are commanded. The second iteration is the same as the first iteration except that ANN I does not need to be retrained.

Figure S5 shows the distribution of the cumulative distribution function (CDF) (a) and probability density function (PDF) (b) with different Ti content in A356-0.005Sr in the first iteration of Bayesian optimization. And they correspond

to $\Phi(x)$ and $\phi(x)$, respectively, in the function of expectation improvement. Obviously, the distributions of both functions are concentrated around a Ti content of about 0.08 wt.%.

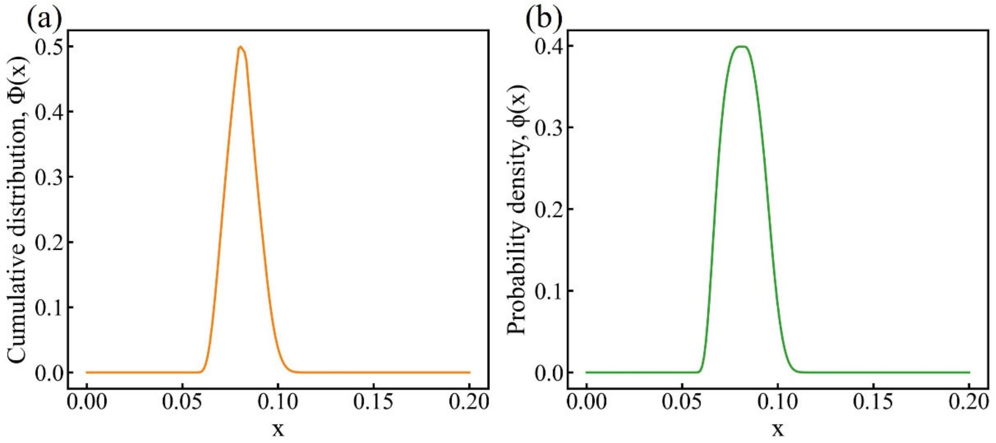

Figure S5. Distribution of the cumulative distribution function (CDF) (a) and probability density function (PDF) (b) with x. The x is equal to the Ti content in A356-0.005Sr.

4. Analysis of microstructure and strengthening and toughing mechanisms

As shown in Figure S6 (a), the average grain size of α-(Al) decreases with the addition of Ti. The main reasons are: (i) as reported by Zhang et al [25], Ti element has a growth restriction effect [25,26] on aluminum alloy, which leads to the reduction of grain size of α-(Al). And Figure S6 (b) shows the calculated growth restriction factor Q_{true} of A356-0.005Sr with different Ti content in this work, which increases with increasing Ti content and is consistent with the results of Zhang et al. It is further demonstrated that one of the reasons for the reduction of the α-(Al) in this work is the growth restriction effect of Ti addition; (ii) it is known from thermodynamic calculations that when a certain amount of Ti element is added to the alloy, Al_3Ti phase is produced in the alloy, which becomes a heterogeneous nucleation point of Al. With the increase of Ti content, the elongation showed a trend of first increasing and then decreasing. This is because a small amount of Ti addition leads to the refinement of grain size of the alloy and the increase of alloy elongation; With the further increase of Ti element, the solid solution of Ti element to α-(Al) further increases, which leads to the increase of yield strength and the decrease of elongation of the alloy. Figure S6 (c) shows the size distribution of α-(Al). At a Ti content of about 0.08 wt.% for the A356-0.005Sr alloy, the alloy size distribution is more concentrated in small sizes. It is consistent with the average grain size of α-(Al) and the mechanical properties of the alloy.

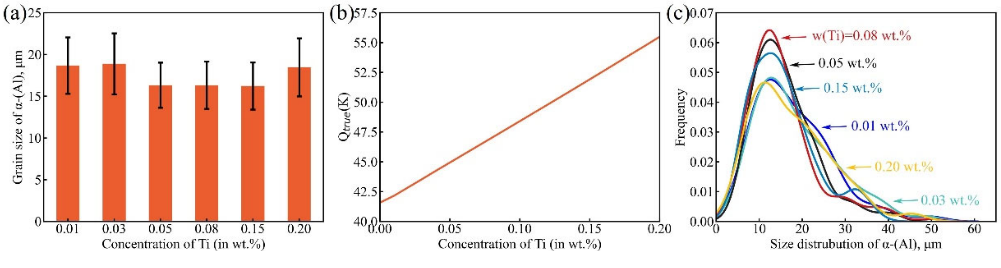

Figure S6. Analysis of strengthening and toughing mechanisms by experiments and computational thermodynamic. (a) the average grain size of α-(Al) in A356-0.005Sr-xTi alloys; (b) calculated growth restriction factor Q_{true} with different Ti content for A356-0.005Sr, (c) the size distribution of α-(Al) in A356 alloys with different Ti additional contents.

5. The second iteration of Bayesian optimization

To further validate the optimal Ti content of A356-0.005Sr, the experimental data from the recommended points were added to the original dataset to form a new experimental dataset for machine learning. Figure 7 shows the distribution of the cumulative distribution function (CDF) (a) and probability density function (PDF) (b) with x. The x is equal to the Ti content in A356-0.005Sr in the second iteration of Bayesian optimization. Obviously, compared with the first

iteration, the distributions of both functions are more concentrated near the optimal value in this iteration, and the Ti content of this aggregation is similar to the Ti content of the last iteration.

The same process as the last iteration was performed and the results are shown in Figure S8. The results of multiple machine learning and experimental mechanical properties are shown in Figure S8 (a). Compared to the previous results, there is a significant reduction in the uncertainty of the machine learning results for mechanical properties. The uncertainty in model predictions decreases with experimental data, which is quite reasonable. Similarly, the quality index and the expected improvement (EI) of the quality index in the A356-0.005Sr alloy with different Ti content were calculated, as shown in Figure S8 (b). And the maximum EI was used to recommend the next point with the potential best mechanical properties for the A356-0.005Sr-*x*Ti alloy, as shown by the black dotted line in the figure. After adding the new experimental data, its recommended Ti content for the alloy with the potential best comprehensive performance is 0.082 wt.%. Obviously, the new point recommended with the Ti content of 0.082 wt.% after adding new experimental data is the almost same as the previous best one. Mechanical properties of alloys are determined by a combination of different factors, where very small differences in composition are almost negligible. Therefore, it indicates that the Bayesian optimization tends to stabilize. And it can be stated that the A356-0.005Sr- alloy at a Ti content of 0.08 wt.% has the best comprehensive mechanical properties.

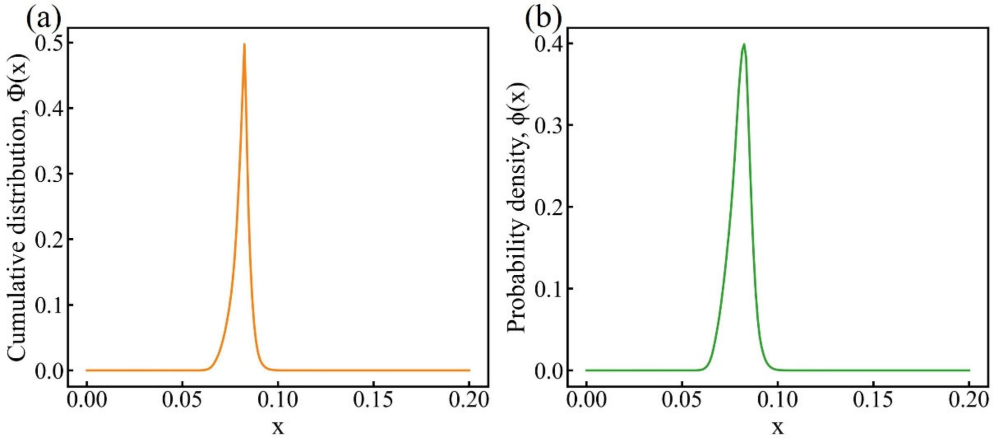

Figure S7. Distribution of the cumulative distribution function (CDF) (a) and probability density function (PDF) (b) with x. The x is equal to the Ti content in A356-0.005Sr in the second iteration of Bayesian optimization.

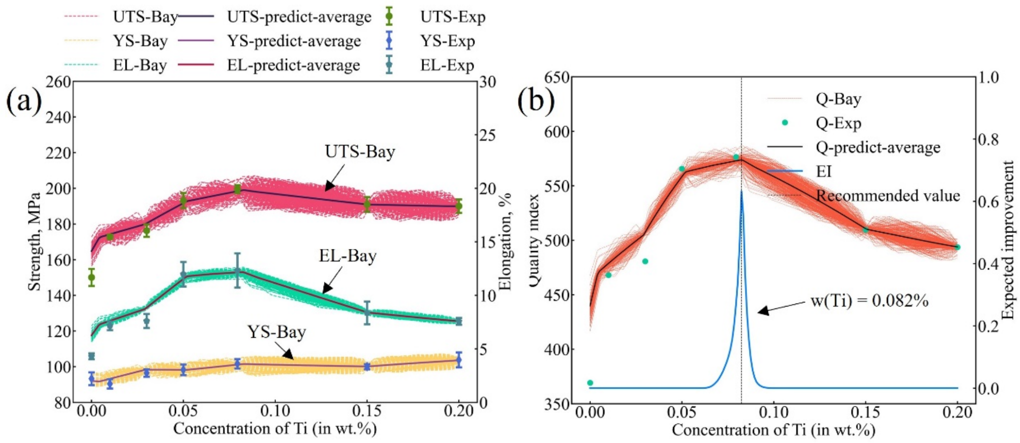

Figure S8. Machine learning results after adding the new experimental data in the A356-0.005Sr alloy with different Ti contents. (a) Simulation results of mechanical properties after adding the experimental values of the recommended point by EI, the solid symbols represent the experimental data (including the recommended point), (b) Quality index, and expected improvement value of A356-0.005Sr with different Ti content computed from the simulation results and experimental data, the black dotted line represents next recommended value by EI.

References

1. Li, Y.; Gu, Q.-F.; Luo, Q.; Pang, Y.; Chen, S.-L.; Chou, K.-C.; Wang, X.-L.; Li, Q. Thermodynamic Investigation on Phase Formation in the Al–Si Rich Region of Al–Si–Ti System. *Mater. Des.* **2016**, *102*, 78–90, doi:10.1016/j.matdes.2016.03.144.
2. Chen, X.G.; Fortier, M. TiAlSi Intermetallic Formation and Its Impact on the Casting Processing in Al–Si Alloys. *J. Mater. Process. Technol.* **2010**, *210*, 1780–1786, doi:10.1016/j.jmatprotec.2010.06.009.
3. Dezellus, O.; Gardiola, B.; Andrieux, J.; Lomello-Tafin, M.; Viala, J.C. On the Liquid/Solid Phase Equilibria in the Al-Rich Corner of the Al-Si-Ti Ternary System. *J. Phase Equilib. Diffus.* **2014**, *35*, 137–145, doi:10.1007/s11669-014-0282-1.
4. Peronnet, M.; Barbeau, F.; Bosselet, F.; Viala, J.; Bouix, J. Comportement Chimique Du Titane Dans Un Alliage Liquide Aluminium-Silicium. *J. Phys. IV France* **1999**, *09*, 223–228, doi:10.1051/jp4:1999429.
5. Dinsdale, A.T. SGTE Data for Pure Elements. *Calphad* **1991**, *15*, 317–425, doi:10.1016/0364-5916(91)90030-N.
6. Tang, Y.; Du, Y.; Zhang, L.; Yuan, X.; Kaptay, G. Thermodynamic Description of the Al–Mg–Si System Using a New Formulation for the Temperature Dependence of the Excess Gibbs Energy. *Thermochim. Acta* **2012**, *527*, 131–142, doi:10.1016/j.tca.2011.10.017.
7. Zhong, Y.; Wolverton, C.; Chang, Y.A.; Liu, Z.-K. A Combined CALPHAD/First-Principles Remodeling of the Thermodynamics of Al–Sr: Unsuspected Ground State Energies by "Rounding up the (Un) Usual Suspects." *Acta Mater.* **2004**, *52*, 2739–2754, doi:10.1016/j.actamat.2004.02.022.
8. Kattner, U.R.; Lin, J.-C.; Chang, Y.A. Thermodynamic Assessment and Calculation of the Ti-Al System. *Metall. Trans. A* **1992**, *23*, 2081–2090, doi:10.1007/BF02646001.
9. Zhong, Y.; Sofo, J.O.; Luo, A.A.; Liu, Z.-K. Thermodynamics Modeling of the Mg–Sr and Ca–Mg–Sr Systems. *J. Alloys Compd.* **2006**, *421*, 172–178, doi:10.1016/j.jallcom.2005.09.076.
10. Yi, W.; Gao, J.; Tang, Y.; Zhang, L. Thermodynamic Descriptions of Ternary Al–Si–Sr System Supported by Key Experiments. *Calphad* **2020**, *68*, 101732, doi:10.1016/j.calphad.2019.101732.
11. Peng, Y.; Zhao, D.; Hu, B.; Zhou, L.; Du, Y.; Gang, T.; Liu, S.; Chang, K. Thermodynamic Modeling of the Sr-M (M= Fe, Mn, Ni, Ti, V) Systems. *J. Phase Equilib. Diffus.* **2010**, *32*, 42–47, doi:10.1007/s11669-010-9818-1.
12. Murray, J.L. The Mg-Ti (Magnesium-Titanium) System. *Bulletin of alloy phase diagrams* **1986**, *7*, 245–248, doi:10.1007/BF02868999.
13. Edalati, K.; Emami, H.; Staykov, A.; Smith, D.J.; Akiba, E.; Horita, Z. Formation of Metastable Phases in Magnesium–Titanium System by High-Pressure Torsion and Their Hydrogen Storage Performance. *Acta Mater.* **2015**, *99*, 150–156, doi:10.1016/j.actamat.2015.07.060.
14. Janz, A.; Gröbner, J.; Mirković, D.; Medraj, M.; Zhu, J.; Chang, Y.A.; Schmid-Fetzer, R. Experimental Study and Thermodynamic Calculation of Al–Mg–Sr Phase Equilibria. *Intermetallics* **2007**, *15*, 506–519, doi:https://doi.org/10.1016/j.intermet.2006.09.001.
15. Kerimov, K.M.; Dunaev, S.F.; Sljusarenko, E.M. Investigation of the Structure of Ternary Phases in Al-Mg-Ti, Al-Mg-V and Al-Mg-Cr Systems.Pdf. *J. less-common met.* **1987**, *133*, 297–302, doi:10.1016/0022-5088(87)90240-2.
16. Wei, Y.; Aiping, W.; Guisheng, Z.; Jialie, R. 5A06/TA2 Diffusion Bonding with Nb Diffusion-Retarding Layers. *Mater. Lett.* **2008**, *62*, 2836–2839, doi:10.1016/j.matlet.2008.01.058.
17. Zhang, F.Y.; Yan, M.F.; You, Y.; Zhang, C.S.; Chen, H.T. Prediction of Elastic and Electronic Properties of Cubic Al18Ti2Mg3 Phase Coexisting with Al3Ti in Al–Ti–Mg System. *Physica B: Condensed Matter* **2013**, *408*, 68–72, doi:10.1016/j.physb.2012.09.039.
18. Wang, J.; Liu, T.; Liu, Y.; Wu, C.; Su, X. Study on Evolution of Ti-Containing Intermetallic Compounds in Alloy 2618-Ti during Homogenization. *High Temp. Mater. Process.* **2015**, *34*, 621–625, doi:10.1515/htmp-2014-0136.
19. Yi, W.; Liu, G.; Lu, Z.; Gao, J.; Zhang, L. Efficient Alloy Design of Sr-Modified A356 Alloys Driven by Computational Thermodynamics and Machine Learning. *J. Mater. Sci. Technol.* **2022**, *112*, 277–290, doi:10.1016/j.jmst.2021.09.061.
20. Dinsdale; A., T. SGTE Data for Pure Elements. *Calphad* **1991**, *15*, 317–425, doi:10.1016/0364-5916(91)90030-N.
21. Redlich, O.; Kister, A.T. Algebraic Representation of Thermodynamic Properties and the Classification of Solutions. *Ind.eng.chem* **1948**, *40*, 345–348.

22. Hao, D.; Hu, B.; Zhang, K.; Zhang, L.; Du, Y. The Quaternary Al–Fe–Ni–Si Phase Equilibria in Al-Rich Corner: Experimental Measurement and Thermodynamic Modeling. *J. Mater. Sci.* **2014**, *49*, 1157–1169, doi:10.1007/s10853-013-7795-6.
23. Zhang, L.; Wang, J.; Yong, D.; Hu, R.; Nash, P.; Lu, X.G.; Chao, J. Thermodynamic Properties of the Al–Fe–Ni System Acquired via a Hybrid Approach Combining Calorimetry, First-Principles and CALPHAD. *Acta Mater.* **2009**, *57*, 5324–5341, doi:10.1016/j.actamat.2009.07.031.
24. Sundman, B.; Ågren, J. A Regular Solution Model for Phases with Several Components and Sublattices, Suitable for Computer Applications. *J. Phys. Chem. Solids* **1981**, *42*, 297–301, doi:10.1016/0022-3697(81)90144-X.
25. Zhang, J.; Gao, J.; Song, B.; Zhang, L.; Han, C.; Cai, C.; Zhou, K.; Shi, Y. A Novel Crack-Free Ti-Modified Al-Cu-Mg Alloy Designed for Selective Laser Melting. *Addit. Manuf.* **2021**, *38*, 101829, doi:10.1016/j.addma.2020.101829.
26. Easton, M.; StJohn, D. An Analysis of the Relationship between Grain Size, Solute Content, and the Potency and Number Density of Nucleant Particles. *Metallurgical and materials transactions A* **2005**, *36*, 1911–1920.
